# Sustainable management of biological solids in small treatment plants: overview of strategies and reuse options for a solar drying facility in Poland

**DOI:** 10.1007/s11356-020-10200-9

**Published:** 2020-07-24

**Authors:** Joanna Boguniewicz-Zablocka, Iwona Klosok-Bazan, Andrea G. Capodaglio

**Affiliations:** 1 Department of Thermal Engineering and Industrial Facilities, Faculty of Mechanical Engineering, Opole University of Technology, Opole, Poland; 2 Department of Civil Engineering and Architecture, University of Pavia, 27100 Pavia, Italy

**Keywords:** Sewage sludge, Sludge disposal, Sludge drying, Biosolids reuse, Sustainability

## Abstract

The issue of sustainable management of biosolids (excess sludge) from wastewater treatment is an important issue in the entire developed world. Residual sludge disposal costs and environmental impact may be significant, and reducing such costs, as well as the energy consumption for dewatering and drying, is a key issue for safe and sustainable sludge disposal, considering the recent ban of some disposal options, such as landfilling, in many European countries. An alternative to thermal technologies is solar drying (not to be confused with bio-drying, very close to the concept of composting). Solar greenhouse drying technology is characterized by reduced land requirements compared with traditional outdoor drying beds, as well as by low-energy requirements compared with other thermal drying methods. Process operation is cost-efficient, with close to no maintenance, and observed specific evaporation rates up to threefold higher than conventional drying beds. Many applications of this technology exist in Poland, Germany and Austria: more than 10,000 t of wet sludge per year is treated in this way in Germany alone and almost as many (9000 t/year) in Poland. This paper examines current biosolids treatment technologies applicable to small wastewater treatment plants (2000–9999 population equivalents served) and opportunities for possible solids reuse in Poland in view of sustainable circular economy schemes. In particular, a purely solar-driven greenhouse facility for sewage sludge drying was investigated under different conditions (season, temperature, environmental humidity) and possible improvements for its efficiency evaluated. Sludge processed by solar drying could have different final disposal pathways, according to season, in accordance with the prescriptions of the new National Waste Management Plan of Poland.

## Introduction

Large quantities of residual biosolids (excess sewage sludge) are produced in wastewater treatment plants (WWTPs) worldwide. Safe disposal of waste sludge is an important issue as at the European Union level, since its production is estimated to exceed 14 million tons (dry weight, d.w.) in year 2020. Disposal costs may be significant, up and above 50% of the operational costs of a WWTP, and may contribute around 40% of greenhouse gas (GHGs) emissions associated with it (Callegari and Capodaglio 2018). Most disposal options rely on specific processes to reduce sludge volume and weight (raw sludge water content is in excess of 97%) and stabilize residual organics and pathogens, which make handling unsanitary and overly expensive. Water content reduction is highly energy demanding, with an efficiency benchmark set by the thermodynamic requirement for evaporation, approximately 0.63 kWh/kg. In practice, depending on original sludge humidity and actual applied technology, this energy demand may be around 2500 kWh/ton d.w. (Capodaglio and Olsson 2020). Reducing energy consumption of sludge dewatering is therefore a key issue for sludge disposal, in view of limitations foreseen for some current options, such as landfilling (Bień and Bień 2015). Other options (e.g. land application), widely common in the past, are limited by metals and other hazardous pollutants content, due to their potential direct or indirect (linked to environmental accumulation) health effects: regulations concerning sludge disposal are getting increasingly restrictive in many countries. Agricultural sludge disposal in Europe decreased from 98.6% in 1985 to 78% in 2015 (Eurostat 2019). Following concerns about harmful compounds build-up in soils following agricultural application, 16 EU members have introduced stricter requirements for metals content, while some, including Finland, Slovenia, Sweden, the Netherlands, Greece and Belgium, have completely prohibited disposal of untreated sludge on soil. Initially regarded as appropriate strategy for improving soil productivity by increasing organic matter and nutrient content, land disposal of untreated sludge is becoming largely unacceptable by public opinion, in consideration of many demonstrated or perceived detrimental environmental effects.

It was estimated that sludge produced in Poland in 2018 exceeded 0.7 Mt, mostly disposed of in agriculture (National Waste Management Plan 2010). This is viewed unfavourably by the Polish Government in terms of commitments to EU Directives and of adverse environmental impact, with alternative sustainable solutions being investigated (Werle and Sobek 2019). Thermal processing of more than 30% of all biologic waste is recommended by the latest Polish National Waste Management Plan (NWMP); however, due to its high costs, this solution is only applicable in large urban agglomerations (Przydatek and Wota 2020).

This paper examines current biosolids thermal treatment technologies applicable to small wastewater treatment plants (2000–9999 population equivalents, PE) and opportunities for solids reuse in Poland under sustainable circular economy schemes. In particular, solar drying of sewage sludge from a small facility was investigated under different operating conditions in order to assess this process’ limitations. Due to the relative technological simplicity of greenhouse drying, and of its adoption mostly in small facilities, this is seldom addressed by detailed investigations; however, its contribution to countrywide sustainable biosolids management may be significant, especially in predominantly rural areas. Improved management of biosolids at small facilities could facilitate their inclusion in efficient, regional disposal schemes. Process improvement and sludge final disposal options in accordance with the prescriptions of new National Plan are also discussed.

## Properties of biosolids

Properties and quantities of WWTPs’ residual biosolids are closely linked to the characteristics of the contributing catchment and of the treatment facility. The latter, in particular, has a considerable influence on their production: aerobic processes, with faster kinetics, generally produce greater amounts of excess biomass, with some notable exceptions (i.e. granular aerobic sludge, membrane processes); slower anaerobic processes kinetics (i.e. UASBs, EGSPs) generate less. It was estimated that the energy required for sludge disposal ranges between 50 and 100 kWh/m^3^ wastewater treated in aerobic facilities, compared with 5 kWh/m^3^ in anaerobic ones (Bruce and Fisher 1984), and this is one of the many reasons prompting the latter’s adoption under new urban sewerage sustainability paradigms (Zeeman et al. 2008; Capodaglio et al. 2016b; Capodaglio 2020). Other microbially based unconventional processes, such as bioelectrochemical processes, may generate even less waste sludge (Callegari et al. 2018a).

Biosolids on average contain 20–41% carbon, 1.5–5% nitrogen and 0.2–11% phosphorus (d.w.), making them a potentially interesting substrate for nutrients and/or energy recovery (Daneshgar et al. 2019). However, they may also contain variable amounts of metals and organic contaminants, including chlorinated and perfluorinated compounds, biphenyls, hydrocarbons, antibiotics and pharmaceuticals, quaternary ammonium compounds, steroids and others. In addition, they may contain undesirable microorganisms, pathogenic (*Salmonellae*) and not (e.g. *Escherichia coli*, faecal coliforms, *clostridia*, *coliphages*). All these are key issues limiting biosolids reuse and recycling options.

Excess sludge is also a chemical energy concentrate. Wastewater organics embed an energy content estimated at 3.86–8.3 kWh/kg COD_oxidized_, the range representing different measurement procedures (Capodaglio and Olsson 2020). Sludge volatile-to-total solids ratio is about 5 times higher than raw wastewater’s (around 0.10), with proportionally higher specific embedded energy. For this reason, sewage sludge is looked at with increasing interest as feedstock for the production of renewable fuels (Capodaglio and Callegari 2018).

### Biosolids sustainable disposal

An important step towards environmental sustainability and practical implementation of water cycle circular economy can be achieved by strategies aiming at the maximization of the value of excess biosolids, at the same time minimizing cost and environmental impacts throughout their life cycle. Simple, safe and cost-effective management alternatives should stress locally compatible approaches for reclamation of resources. A sludge disposal technology, to be considered sustainable, should generate a stabilized and disinfected final product with added value, easy to handle, while being easy to maintain and operate.

As mentioned, the most important beneficial uses of municipal biosolids involve the exploitation of embedded energy and resources (mostly nutrients). Land application of untreated biosolids, a common practice in the past, is currently frowned upon, if not outright banned, in most situations due to potentially adverse impacts. In order to limit those, thermal processing of sludge remains perhaps one of the most straightforward approaches for disposal of waste sludge without excessive secondary pollution and risks, allowing some forms of energy and resources recovery (Capodaglio et al. 2016a). Until a few years ago, incineration was one of the few technologies believed to adequately deal with sludge not suitable to agricultural use (Englande and Reimers 2001). Recently, progresses in processes such as pyrolysis, hydrothermal carbonisation (HTC) and gasification allow transformation of biosolids into biochar or hydrochar form (Bolognesi et al. 2019) or syngas (Werle and Sobek 2019).

Some of these approaches, readily implementable in medium-/large-scale wastewater treatment facilities, may however be too challenging for small plants, such as those of many small towns in the Polish countryside, a large fraction of the over 3200 operating in the country, most of them biological (with about a quarter of them designed for nutrients removal) (Przydatek and Wota 2020). Only a small amount (estimated at < 30%, short of NWMP targets) of sludge produced in Poland is thermally utilized today, with large amounts (almost nine-folds the annual production) left accumulating on landfill areas near WWTPs (Werle and Sobek 2019). Polish law changes following EU Directives are prompting new approach paradigms and improved technologies for sludge disposal (NWMP-2022 2016).

### Biosolids dewatering and drying

Sludge dewatering and drying are fundamental steps in any sustainable sludge management strategy. The former must not be confused with the latter: one reduces free water content so that solids in sludge reach about 20%, allowing its handling like a semisolid material. This can be achieved mechanically using filter/belt presses, centrifuges, geomembranes, open drying beds and other processes. Drying, on the other hand, is normally achieved through thermal processes evaporating bound water to reach a final solids content of about 90%. Recent technologies combine dewatering and drying in a single step. Drying significantly reduces sludge environmental impact, producing a stabilized, dry granular product more easily transported and better suited for agricultural use (where permitted) since nutrients are mostly retained within and other problematic components are eliminated/immobilized. The drying process also adds value to the residuals, enhancing the efficiency of many reuse and recovery technologies.

## Thermal treatment and resource recovery

Among thermal treatment options, incineration is considered a safe technology for energy recovery from waste and sludge, although sometimes viewed by public opinion with criticism, due to atmospheric emissions and hazardous residues issues. Pyrolysis, a thermochemical decomposition in the absence of oxidizing agents, is also increasingly used. This process rearranges chemical bonds of organic molecules, producing residual solid, gaseous and liquid fractions with fuel and/or material value. Pyrolysis can be achieved with energy input from different sources (fossil, electric, radiation) in different operating conditions (Callegari et al. 2020). Gasification, a thermochemical decomposition in an oxygen-depleted environment, is used to maximize gas production (Werle and Sobek 2019).

Thermal processing is energy-intensive: the large quantities of water in sludge (primary sludge contains around 93–97%, secondary 98–99%) severely limit its recovery efficiency. Unless initial sludge humidity is below 30%, more energy is required for drying than is generated by combustion (Callegari et al. 2018b). The breakeven point for energy recovery from sludge combustion was determined to be 27% water content; however, the use of dry sludge (> 90% solids) is normally considered more appropriate to successfully recover its energy content (Andriessen et al. 2019). Mechanically dewatered sludge cannot be incinerated autothermally (at process temperature > 850 °C) without auxiliary fuel, with high operational cost. Energy-positive pyrolysis occurs at feedstock humidity below 10% (Raček et al. 2017). Pyrolysis and gasification, unlike incineration, generate forms of storable energy (e.g. bio-oil, biochar, syngas) and other residuals suitable to feed circular economy circuits.

Emergy assessment could help to identify the most sustainable processes to achieve economic and social benefits with minimal environmental impact. Emergy analysis is a method of accounting materials’ embodied energy by expressing all the considered streams and processes in solar energy equivalents. It can then be used to compare different biosolid management alternatives. An emergy-based sustainability assessment of biosolids disposal options confirmed that gasification is energetically and environmentally suitable for biosolids disposal, and pre-processing of biosolids prior to their use in agriculture was shown to improve sustainability performance of this option (Cano Londoño et al. 2017).

The use of thermal drying as pretreatment prior to final residuals disposal is encouraged and increasing. This allows volume, weight and transportation costs reduction and inhibition of biochemical reactions that may occur in the presence of excess residual humidity, allowing safer storage and contamination hazards reduction. In practical applications, depending on technology, drying requirements range from 0.82 to 1.1 kWh/kg sludge, with innovative combined processes claiming values as low as 0.24 kWh/kg (Shincci Energy 2019). Despite the increasing diffusion of drying processes, their application is still economically and technically challenging. Energy for evaporation can be supplied by electric, fossil or microwave sources through conduction, convection, radiation and other processes, requiring high temperatures and constant airflow to extract saturated vapour from substrate. Main thermal drying technologies are tunnel or rotary drying (Chen et al. 2015; Treviño Arjona and Rodríguez Cisneros 2005). Energy requirements of current drying technologies are summarized in Table 1.

**Table 1:** Energy requirements of current biosolids drying technologies

Technology	Energy (kWh/L_water_)
Low temperature spiral press/evaporator^a^	0.24
Band dryer	0.82
Paddle dryer	0.95
Flash dryers	1.05
Rotary dryers	1.05
Drum	1.07
Fluid bed	1.10

^a^According to (Shincci energy 2019)

### Recoverable energy and products from dried biomasses

Pyrolysis generates liquid (a.k.a. bio-oil, py-oil), solid (biochar) and gaseous (syngas, py-gas) recoverable phases; gasification produces syngas and solid residues. Py-oil is a liquid product similar in composition to biodiesel from energy crops and diesel fuel, although with higher content of impurities. Its low calorific value (LCV) is lower than energy crops biofuel, 33–35 MJ/kg, against those from corn and safflower (42–43 MJ/kg) and of fossil fuel (~ 30% lower than diesel), depending on process conditions (Capodaglio and Dondi 2016).

Biochar is a “porous carbonaceous solid produced by thermochemical conversion of organic materials in oxygen depleted atmosphere that has physicochemical properties suitable for safe and long-term storage of carbon in the environment” (Shackley et al. 2012), with energy value of 15–18 MJ/kg, depending on feedstock and process. It could be burned for energy; however, it has significant other uses, e.g. improvement of soil productivity (carbon and nutrient content), remediation of contaminated soils (high adsorption potential) and mitigating effects of climate change (long-term carbon fixation). Researchers have shown that biochar can minimize metals release, lowering environmental impacts as soil improver, compared with untreated sludge (Racek et al. 2019).

Syngas is primarily composed of hydrogen and carbon monoxide, with smaller quantities of methane, carbon dioxide, water and low molecular-weight volatiles. Its heating value may vary according to process conditions (normally about 6 MJ/kg in pyrolysis, higher in gasification).

It should be remembered that recovery processes of all these products are energetically sustainable when feedstock sludge humidity is below about 10%; however, the energy required to initially dry sludge to that level must also be included in a process’ sustainability analysis.

### Polish biosolids disposal perspective

The number of biological WWTPs in Poland increased in recent years, serving a greater share of previously unconnected population, with dismission of older facilities based only on mechanical treatment. Sewage sludge production has thus considerably increased, with a general lack of facilities for thermal disposal. While in large urban areas sludge is converted into energy in heating or power plants, smaller cities are left with fewer choices. There are currently only about 30 thermal sludge drying installations in Poland, based mostly on belt dryers using natural gas as energy source, servicing large (> 99,000 PE) municipal WWTPs. Due to the scarcity of disposal facilities available, thermal disposal is more expensive than average elsewhere the EU, as shown in Table 2, comparing EU and Polish disposal costs for various process options based on standard dewatered sludge (~ 80% humidity) as base feedstock (Kacprzak et al. 2017). This partly explains local sludge management choices, where agricultural, soil amendment and composting comprise about 50% of final destinations, while thermal still remains below 30% (Statistics Poland 2019). In small-medium size plants, there is hence ample margin to increase the efficiency of energy recovery from sludge residuals. Given the largely rural economy of the country, the most interesting recovery options lie especially in sustainable soil-oriented applications, including nutrient (phosphorous) recycling, a finite resource (Daneshgar et al. 2018a).

**Table 2:** Cost comparison of average sludge disposal options in Poland and the EU (based on (Kacprzak et al. 2017))

Method of disposal	Polish cost (Euro/t)	Average EU cost (Euro/t)
Agriculture	75	160-210^a^
Forestry	75	240
Composting	150	310
Incineration^b^	375-438^c^	315
Landfilling	125	255

^a^Higher figure refers to dried sludge

^b^Excluding cost recovery from produced energy

^c^Co-incineration and mono-incineration, respectively

Gasification is a popular technology in Poland, mostly associated with the countrywide diffusion of agricultural biomasses conversion plants. Incidentally, it should be noted that the residual solid fraction of the biomass gasification process consists of over 20% P_2_O_5_ (natural phosphate rock contains about 28%) and that studies showed > 73% efficiency of phosphorus recovery from gasification solid residuals leaching (Gorazda et al. 2017). Co-combustion with fossil fuels (coal, lignite, wood) and municipal solid waste is also considered an interesting option, as it requires little or no additional investment to add small quantities of sludge the mass of burned fuel. However, two obstacles undermine the wider implementation of such methods in Poland: the first is public acceptance, and the second is purely economic. Public concern and prejudice against combustion plants must be properly addressed by institutional support strategies and incentives (Capodaglio et al. 2016c). According to the NWMP-2022, thermally treated sewage sludge fraction should increase substantially in coming years.

## Solar drying of biosolids

An alternative to energy-intensive thermal technologies for sludge drying is the use of free solar energy. Solar drying is an effective method of applying solar radiation with the aim of eliminating activity of microorganisms and excess humidity content to many agricultural products and organic process residuals, including sludge. Among the various existing solar dryer types, greenhouse dryers have numerous advantages over others, making them a good process alternative for excess sewage sludge processing (Singh et al. 2018). The aim of the process is to accelerate water elimination from the substrate by exploiting an artificial indoor environment in which vapour pressure equilibrium between sludge and ambient air is inhibited by forced ventilation. Solar drying is not to be confused with bio-drying, an alternative method aiming at water removal using heat generated by aerobic organics degradation, conceptually very close to composting (Bennamoun 2012).

These systems are based on an improved greenhouse scheme: concrete flooring with integrated drainage and enclosure by transparent covering (plastic foil, glass or Plexiglas) allow high transmittance of incoming radiation. The impermeable floor surface is profiled with small channels in such a way that free excess water is collected, flowing to a collection sump well, from where it can pumped back into the liquid process units. However, since the drying sludge has already been dewatered prior to layering into the greenhouse, there is normally little free water collected by the drains. Mechanical sludge mixers support moisture transfer from the bulk of the sludge to the surface in contact with the drying air: as shown by Krawczyk (2019), adequate sludge bulk mixing is more efficient than operation at reduced sludge layer thickness. Mechanically, dewatered sludge is spread in layers of 20–50 cm; frequent mixing and continuous aeration ensure odour-free and efficient drying. The structures are fitted with exhaust air fans and ventilation louvers to control air exchange and introduce fresh air, optimizing the drying potential of the indoor environment. Suspended, horizontally mounted fans provide air turbulence on the sludge surface to enhance humidity removal rates. The main liquid transfer phenomena occur at the surface boundary layer, at a rate *W* be expressed by the following:

1 $$ W=A\cdotp {k}_y\ \left({Y}_s-Y\right) $$

where *A* is the surface area, *k*_y_ the mass transfer coefficient and *Y*_s_ and *Y* the moisture content in saturated air and in the main air flow, respectively (Krawczyk 2016). From Eq. (1), it can be seen that water evaporation is driven by the air moisture gradient and by the mass transfer coefficient value, mainly depending on the linear velocity of air. All relevant parameters (temperature of sludge and air, relative humidity of internal and ambient air, radiation, wind speed and sludge moisture content) are continuously measured by automated control systems to maximize process effectiveness.

These systems show low specific energy consumption (as low as 0.28 kWh/kg evaporated water for nonflocculated sludge, 0.22 kWh/kg for flocculated sludge), favourably comparing with the 0.82–1.1 kWh/kg normally required by thermal drying (as shown in Table 1). About half of this requirement is due to the ventilation system operation. In warmer countries, with temperatures in the range 26-47 °C, open-airflow greenhouses may be more efficient than climate-controlled ones, without energy requirements except for sludge turning (Belloulid et al. 2019).

Solar drying has shown additional effects on sludge characteristics and composition, causing increase of humic-like substances, decreasing sludge lipids content by complexification of organic matter, favouring its application for long-term organic amendment in land farming (Collard et al. 2017). Sludge overturning frequency can improve overall process efficiency, similarly to what seen in composting and bio-drying (Zhao et al. 2010). A study on open-air drying in warm climate (Arizona, USA) showed that solar drying could promote nitrogen loss via ammonia volatilisation. Dried sludge showed relatively stable organic nitrogen content, with declining ammonium-nitrogen (NH_4_^+^-N) content proportional to moisture loss (O’Shaughnessy et al. 2008).

As part of the solar drying process, odours and pathogen issues may be curtailed. A study in Australia showed reduction of viruses, helminths and bacterial indicators (*Salmonella* sp. and *E. coli*). Results for bacterial pathogens (i.e. *faecal coliforms*) were however inconclusive (Shanahan et al. 2010). A study in Turkey showed that solar drying achieved 1-log reduction of coliform counts within a 45-days, which increased to 4-log reduction in just 5 days by addition of 15% (by weight) lime (Salihoglu et al. 2007); under prevailing Polish climatic conditions, however, solar drying of sludge may not guarantee a product void of pathogenic microorganisms for process times shorter than 4 weeks (Sypuła et al. 2013).

Solar drying process output is a product in granular form (1–5 mm) or dust; apart from higher specific calorific value (≈ 8 MJ/kg) compared with raw sludge, this material has good transportability characteristics (bulk density ≈ 700 kg/m^3^) (Garanto 2016).

Solar drying technology is marked by reduced surface requirements compared with outdoor drying, as well as by low-energy requirements compared with thermal drying. These dryers are popular in small WWTPs, with many existing applications in Germany, treating more than 10,000 t of wet sludge per year, and Austria, countries with climate similar to Poland, where about 9000 t/year is treated annually in this way. Processing is considered cost-efficient, with little maintenance and observed specific evaporation rates up to threefold higher than conventional drying beds (Bux et al. 2002). Its use, however, is limited to small installations as it cannot handle large quantities of sludge due to slow process and low applicable loading rates.

## Solar drying application: case study

This case study addresses the effectiveness of sewage sludge solar drying and possible options for its sustainable final disposal. The WWTP (3200 PE) generating the sludge is located in Ujazd, Opolskie Voivodeship. The plant was built in the 1990s and produces between 300 and 400 t/year of raw sludge, with > 98% moisture content. The sludge undergoes preliminary conditioning (thickening and filtration pressing) that removes up to 9–12% humidity. Until 2013, when the practice was banned by law, press-filtered sludge was stored together with municipal solid waste after stabilization (liming) in open-air heaps. The greenhouse solar drying facility was built in 2014 to reduce sludge volume and improve its management. Although in the south-western part of Poland climatic conditions are not ideally suited for such solutions, the choice of process was primarily due to its low initial economic investment. After a few years of operation, however, performance showed some limits on expected targets. Dried sludge is so far used mainly in agriculture, and only partly in co-combustion facilities, due to their low humidity (< 10%) gate requirement. The issue of sustainable final disposal still remains partly unsolved, as agriculture can currently adsorb only a limited quantity of the production, and specifications for co-combustion are not always consistently achieved. In order to better understand the process and improve its effectiveness, a prolonged monitoring and characterization study was carried out.

**Fig. 1:**
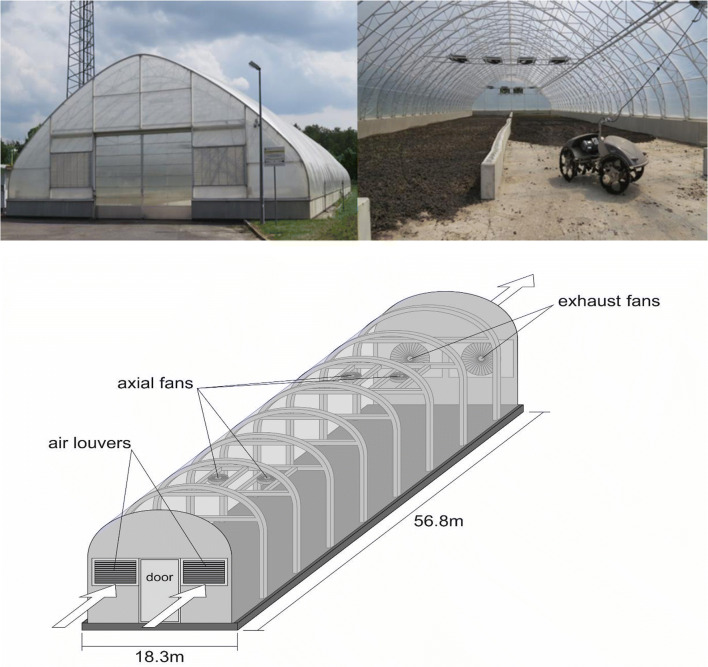
**a** Solar drying facility exterior. **b** Interior, suspended fans and automated turning robot. **c** Schematic view of the drying facility

The unit consists of a free-standing structure, with area of 995 m^2^, 8 m height and 37° roof slope (Fig. 1a). A concrete platform supports the steel frame with high transmittance, polyethylene foil cover. The drying house is equipped with 8 low-speed horizontally suspended fans (1 kW each) to mix air internally, and a cross-ventilation system, consisting of two tilting louvers (6 m^2^) coupled with four exhaust air fans (1 kW each) on the opposite wall and controlled by inverters, for air exchange. Average air velocity in the greenhouse is controlled at about 3 m/s. To achieve better process efficiency, it is necessary to overturn the sludge frequently, which is done by an automated robotic mole (rated power 4.5 kW) (Fig. 1b). Mixing also prevents odour formation and release. A schematic view of the drying facility is presented in Fig. 1c. A control programme determines optimal process conditions by monitoring internal and external environmental parameters (air temperature, air humidity, solar radiation and wind speed) with an array of sensors and adjusting the operation of ventilation, aeration hatches and mixing robot. These are the main energy demands by the process, with total installed power of 16.5 kW. Average annual total power requirements can be assessed as about 0.5 kWh/kg water removed, about 20% less than the theoretical thermodynamic requirement for thermal evaporation (and 39–55% less than most thermal technologies), with total annual energy consumption between 130 and 180 MWh, depending on sludge quantity.

Sludge is towed to the facility on a trailer, manually unloaded, and spread into a uniform layer. After that, the entire drying process occurs in automated mode without manual intervention, which is only required to remove the dried sludge. In 2019, about 35 t of dry solids were processed, at a specific load of approximately 35 kg/m^2^/year. Standard operation assumes that in summer, when sludge water evaporation is faster, layers should be laid 10–20 cm deep, while in winter, the bed thickness could increase to 30–40 cm. Faster summer drying allows processing of a 20-cm sludge layer in about 4 weeks, while in the winter, it takes approximately twice to dry a 30-cm layer. The drying process occurs sequentially in batches, with faster summer process time compensating for increased sludge accumulation in winter. Irradiation, mixing and internal climate control allow seasonally variable degrees (75–91%) of drying and a relatively homogeneous final product for each batch.

Table 3 reports the characteristics of the incoming dewatered sludge during the study. Heavy metals are present at acceptable levels for disposal in agriculture according to Polish regulations.

**Table 3:** Characteristics of dewatered secondary sludge from the experimental campaign

Parameter	Unit	Value	Limit for agriculture use*
pH	-	6.4 ± 0.2	> 5.6
Pathogens count	count/kg DS	0	0
*Salmonella*	count/100 g	Not detected	Not detected
Dry solids	%	93.4 ± 1.3	-
Organic C	% DS	63.0 ± 2.7	-
N-NH_4_	% DS	0.08 ± 0.02	-
Total N	% DS	4.11 ± 0.99	-
Total P	% DS	1.59 ± 0.32	-
Ca	% DS	3.56 ± 0.61	-
Mg	% DS	0.45 ± 0.8	-
Cr	mg/kg DS	14.5 ± 2.5	500
Zn	mg/kg DS	922 ± 157	2500
Cd	mg/kg DS	1.53 ± 0.26	20
Ni	mg/kg DS	14.9 ± 2.5	300
Cu	mg/kg DS	178 ± 30	1000
Pb	mg/kg DS	21.8 ± 3.7	750
Hg	mg/kg DS	< 0.25	16
Calorific value	MJ/kg	14 ± 0.45	-

*Legal limits for agricultural use in Poland based on applicable law (Law Journal of Environmental Ministry 2015)

### Operational monitoring

In order to assess process performance under different operating conditions, monitoring was carried out during two periods: the first started in the cold season (period I, 21 weeks, autumn-winter 2018) and the second in the spring-summer 2019 (period II, 12 weeks). Due to slower evaporation in period I, sludge sampling was performed every 3 weeks, whereas in the spring-summer sampling occurred every 2 weeks. Each time, multiple samples of about 0.2 kg each were collected at random points through the entire depth of the sludge bed with an Egner stick. These were then mixed and a representative cumulated sample extracted for analysis. To minimize measurement errors, the procedure was performed in quadruplicate for each final sample. Collection of incoming sludge samples occurred before and after press dewatering and after drying. Samples were dried at 105 °C for 12 h and desiccated; 44 samples (in quadruplicate) were collected in total during the study.

**Fig. 2:**
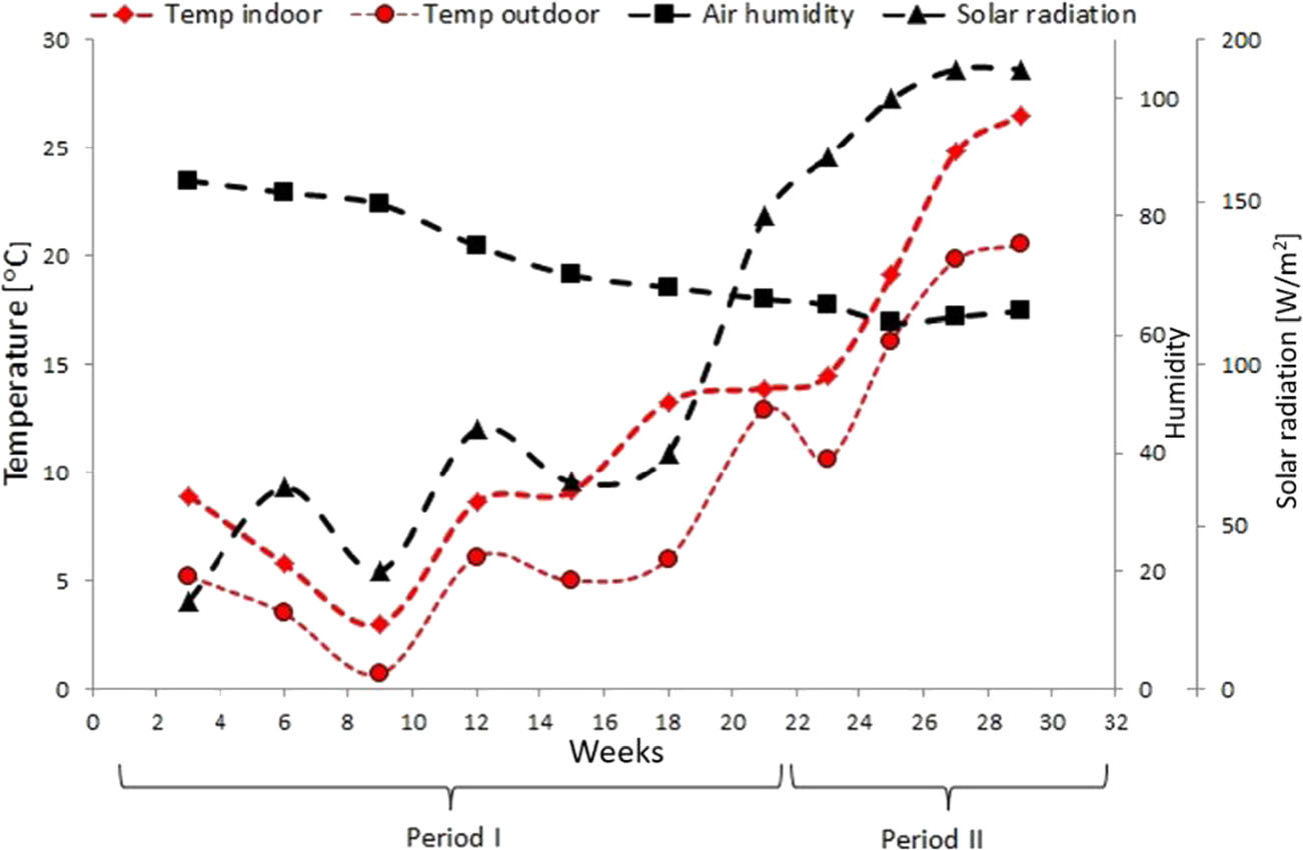
Indoor and outdoor air temperature, solar radiation and humidity during monitoring period

Climatic conditions were monitored outside and inside the greenhouse by the facility’s indoor sensors and external weather station, continuously recording internal and external air temperature, indoor and outdoor humidity, solar irradiation and external wind speed. The mean temperature outside the greenhouse ranged from 0 to 15 °C during study period I and from 10 to 25 °C in period II. Average values of recorded parameters are shown in Fig. 2.

Indoor temperature remained about 3° higher than the external one during period I. In general, low insolation was recorded in winter with lowest (daily) irradiation of 25 W/m^2^, highest in June (187 W/m^2^). Relative average humidity varied considerably, from 60% in February to 82% in May, slightly lower than outdoor humidity, fluctuating from 64% in May to 87% in December. Large infra-day value fluctuations were observed, e.g. in December in the daily range 70 to 90%, in May from 35 to 75%.

**Fig. 3:**
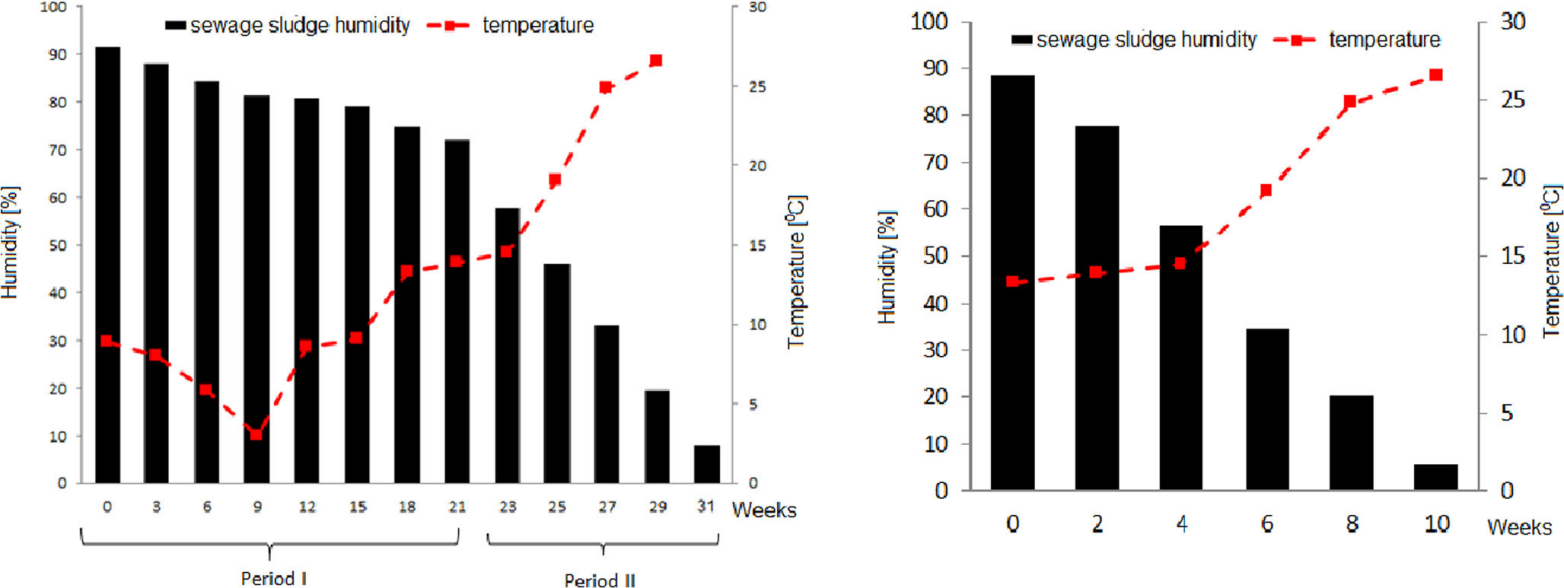
Sludge humidity during study period. **a** Batch 1 (periods I and II). **b** Batch 2 (period II)

The first processed batch, with sludge layered at 30 cm height, was monitored from November to June until drying reached 91.94%. In April, a second batch was layered at 20 cm depth in a separate area of the greenhouse and monitored for 10 weeks. Results are summarized in Table 4. Humidity of the first sludge batch decreased from 91 to 72% (Fig. 3a) in 21 weeks during period I. In period II, it further reduced from 72 to 8%. The second batch humidity decreased over 80% (Fig. 3b) in 10 weeks from 88 to 5.5%. As expected, during winter, when the outdoor average temperature range is 0–6 °C, the process works at lower efficiency. From November to March (period I) the sludge reduced its water content by only 19%. When the average air temperature increased above 12 °C, sludge drying rate increased almost instantly. In these conditions, solar drying allows to consistently obtain over 90% dry solids in 10 weeks or less.

**Table 4:** Results of sludge solids monitoring

		Wet sludge mass (g)	Dried sludge mass (g)	Dry solids (%)	Humidity (%)
Raw sludge		10.16	0.19	1.89	98.11
Dewatered (press) sludge		10.40	0.97	8.56	91.44
**Date**	**Week**	**Batch 1—drying house sludge (periods I and II)**			
23.11.	0	10.04	1.19	11.84	88.16
14.12.	3	10.12	1.60	15.49	84.22
04.01.	6	10.13	1.89	18.64	81.36
25.01.	9	10.04	2.00	19.91	80.78
15.02.	12	10.16	2.11	20.76	79.24
07.03.	15	10.42	2.62	25.14	74.86
28.03.	18	10.19	2.84	27.82	72.18
18.04.	21	10.95	4.63	42.29	57.71
02.05.	23	10.39	5.59	53.83	46.17
16.05.	25	10.31	6.91	66.98	33.02
30.05.	27	13.50	10.84	80.33	19.67
13.06.	29	16.62	15.26	91.94	8.06
		**Batch 2—drying house sludge (period II)**			
04.04.	0	10.96	1.29	11.72	88.28
18.04.	2	10.35	2.33	22.50	77.50
02.05.	4	10.19	4.43	43.45	56.55
16.05.	6	9.97	6.53	65.47	34.53
30.05.	8	10.37	8.27	78.76	20.24
13.06.	10	10.41	12.67	94.47	5.53
		**Batch 3—drying house sludge (period III)**			
17.06	0	10.02	1.19	11.88	88.12
24.06	1	9.95	7.46	64.34	35.66
01.07	2	10.22	11.93	90.74	9.26

A third batch of sludge was layered (20 cm depth) and monitored after period II (period III, June–July). During this period, at average air temperature > 20 °C, sludge drying was fast and humidity reduced by 78% in just 4 weeks, from 88 to 9.2%.

### Results discussion

**Fig. 4:**
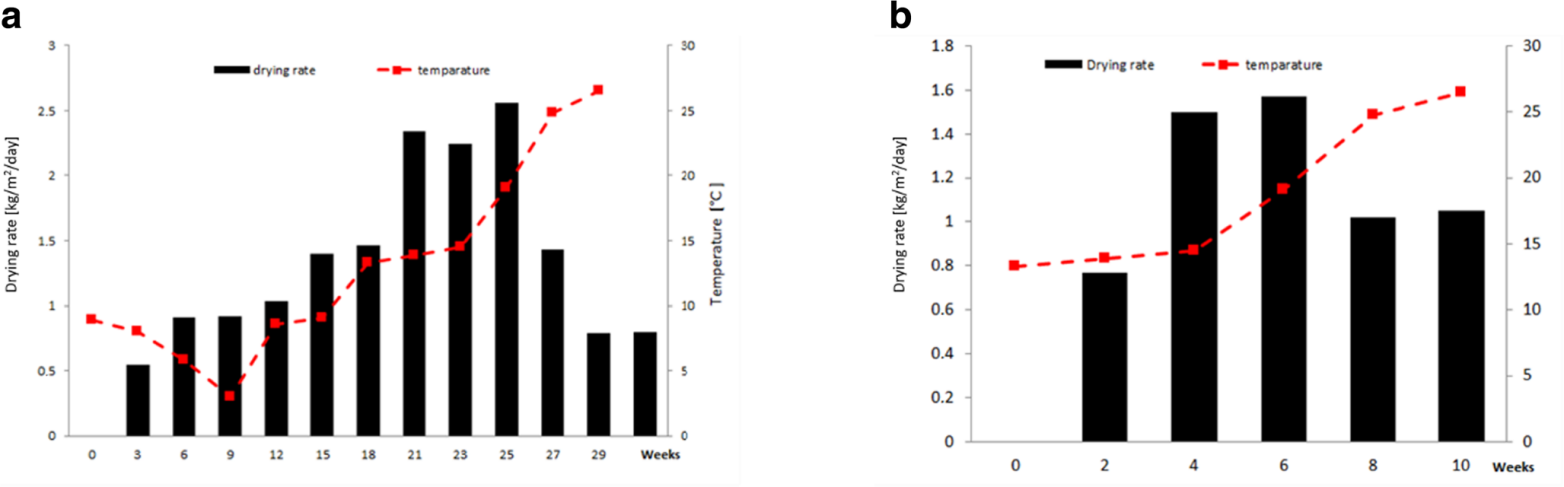
Calculated drying rates during study period. **a** Batch 1 (period I). **b** Batch 2 (period II)

To determine the evolution of the process, sludge drying rates were calculated during periods I and II, as the difference between the initial and final sludge mass at each sampling interval (21 or 14 days), assuming an average sludge density of 977 kg/m^3^. During winter time, up to week 12, with average outdoor temperatures between 3 and 9 °C and air humidity above 70%, average sludge drying rates for batch 1 ranged between about 0.55 and 1.03 kg/m^2^ per day. With temperatures between 10 and 14 °C, and humidity between 65 and 70%, drying rates increased up to 1.46 kg/m^2^/day. With outdoor air temperature over 15 °C and humidity below 65%, drying rates ranged from 1.5 to over 2.5 kg/m^2^/day, an excellent result considering that in this latter phase interstitial and surface water elimination takes place. Initial humidity of batch 1 sludge was 88.16%, final 8.06%, at an average reduction of 2.6% per week. Figure 4 shows the average daily drying rates calculated for the two test periods. For sludge batch 2 (period II), initial drying rate was 0.77 kg/m^2^/day at outdoor temperature 12–14 °C, increasing up to 1.57 kg/m^2^/day with temperature between 15 and 20 °C and remaining above 1 kg/m^2^/day in the latter 4 weeks (outdoor temperature > 20 °C), starting with a residual humidity below 20%. Batch 2 drying process, starting at 88.28% and ending at 5.53% sludge humidity averaged 8.3% drop per week.

Sludge batch 3 (outdoor air temperature > 20 °C, humidity about 60%) showed initial drying rate in the first week of > 50%, decreasing in the second week to about 26%. The average drying rate (starting humidity 88.12%, ending 9.26%) was close to a staggering 40% per week.

While the final moisture content of processed sludge remained below 10% for all batches, process rate varied significantly during the year, requiring over 5 months to achieve the desired results in winter. Although in the tested conditions the drying effect fulfilled expected requirements, it should be considered that wintertime rates are quite low and that consistent achievement of desired targets could be stressed by a foreseeable future increase of sludge production due to WWTP and sewered area expansions. It is thus necessary to implement solutions that could accelerate the process, are environmentally friendly, cost-effective, easily manageable and sustainable.

### Considerations for process improvement

Several proposals have been put forward to improve greenhouse dryers performance, including the improvement of insulation; use of modified geometry (i.e. opaque or inclined northern walls) to prevent heat losses and maximize radiation collection, or of internal thermal storage, to improve heat accumulation during limited solar irradiation periods; and improvement of the systems’ energetic efficiency by integrating photovoltaic panels or of solar air heaters in greenhouse roofs (Singh et al. 2018).

In view of the study results, possible solutions that could improve rates and efficiency of the process were examined, besides the obvious one of expansion of the drying house, which would not improve process rates but just handling capacity. In order to maintain equal duration of drying periods in winter and summer, or at least reduce winter process time by at least 30%, volumetric or underfloor heating and/or infrared lamps to increase sludge irradiation were considered, but these would imply both significant initial investment and energy costs, reducing the current attractiveness of solar drying. A specific study on the economics of solar sludge dryers based on 10 years of Polish experiences determined that, due to climatic conditions, it would be technically impossible to implement a hybrid solar/heated system, operating at a constant rate in each month of the year. Two main reasons are as follows: first, no treatment plant in Poland can count on such excesses of generated energy, especially in winter, when any amount of biogas is needed to heat digesters. Second, the significant solar energy deficit could not be compensated by heat pumps recovery, as shown by Cecconet et al. (2020) for use in residential buildings, due to the limited relative capacity of sewer mains or secondary settling tanks from which heat could be extracted and to the maximum possible temperature of the heating medium (50–55 °C). In addition, even if technically possible, this solution would be unsustainably expensive (Trojanowska 2016). Furthermore, additional energy inputs are unlikely to justify any possible gains from process acceleration, as confirmed by experiences in sludge drying greenhouses serving WWTPs in the Polish towns of Klodzko (PE ~ 30,000) and Myszków (PE ~ 40,000), in similar climatic conditions (Bień 2012).

The effectiveness of dynamic adjustment of sludge bed depth according to season was evaluated, under the example of a similarly built and operated facility at the sewage treatment plant of Żagań (Bień and Bień 2015). In that case, bed thickness was set first at 40–45 cm, achieving minimum sludge solids > 60% in winter. To compensate for lower irradiation, the process was converted to thin layer (10–20 cm) operation in a shorter winter drying cycle of 18 weeks, achieving sludge 73% dry solids in December and 67% in January, showing insignificant improvement over the previous condition.

Mechanical improvement of the sludge cake structure could improve drying capacity by promoting particles agglomeration that would strengthen the skeletal structure of the bed, which could favour water elimination. While the use of physical/chemical conditioners alone may enhance dewatering, but not substantially drying efficiency, a study indicated that mixing locally available clay minerals (bentonite, attapulgite, mixed clays, and zeolite) showed beneficial effects in sewage sludge stabilization and humidity reduction in a Greek greenhouse. An initially highly dewatered sludge (moisture around 84%) was dried significantly more effectively in tests with these minerals’ addition (Samara et al. 2019). Addition of the minerals showed to increase treated sludge fertilizing value, however, potential environmental impacts (in particular with respect to accumulation of Ni, Cr, Zn and B) as well as possible effects on final disposal options different from agriculture were pointed out. Granulated blast furnace slag, an industrial waste, was also used as sludge skeletal-forming material, forming channels that enhanced the removal of excess water. Slag material did not significantly modify the final properties of the treated sludge that was deemed suitable for applications in construction and agriculture (Ramachandra and Devatha 2020).

Some major advantages of solar drying, compared with thermal, are that the former’s costs and energy consumption are much lower, operation is simple and safe and GHG emissions may be up to seven times lower (Oladejo et al. 2018). In order to maintain environmental sustainability without increasing energy input and emissions, reuse of ambient or waste heat from other sources (i.e. nearby industrial complexes) could be considered, reducing space (area) requirements from three to five times and increasing the capacity of existing facilities (Slim et al. 2008). At the moment, however, no such sources exist in close proximity of this facility.

## Sustainable disposal options according to circular economy principles

The examined facility produces on average 35 t/year of dried sludge. Although at the moment this can be used as a “class A” fertilizer in agriculture/greenery management, taking into consideration heavy metal content (CEC 1986), it is uncertain how long this will still be feasible according to evolving national rules. Local, sustainable non-agricultural uses were thus examined: the dried product could be suitable as a fuel in waste-to-energy production, coal-fired power plants or cement plants without further processing, as long as the required humidity specifications are fulfilled.

Due to the high internal temperature, a cement kiln creates favourable conditions for co-combustion of dried sludge, while improving production sustainability. Environmental benefits of using sludge in cement production include the following: reduction of land degradation, reduction of fossil materials extraction (coal) and of other fossil fuels consumption, incorporation of all non-combustible parts of waste (slags, ashes) in cement and reduction of GHG emissions. Although no nutrient recovery is achieved, sludge co-combustion in cement kilns is considered among the best and most ecological waste-free disposal solutions in Poland, due to the immobilization of heavy metals into the cement and the limitation of emission of gaseous pollutants (Bożym and Bok 2017). In Poland, which has a thriving cement industry sector, this could therefore be an excellent solution to municipal sewage sludge disposal; however, it may also be a logistically complex endeavour. This applies to securing adequate supply, transportation and storage of sludge at the cement plant, making such a solution justifiable only for facilities located in close proximity. Dried sludge from this facility meets the specified requirements for cement plants concerning contaminants content (metals, sulphur, chlorine); however, its seasonal qualitative variability influences its calorific value; hence, it is currently disposed of in this way for less than 3% of its production. The cement plant located in Górażdże, 40-km away, is a potential destination, since it could adsorb 1000 t of sludge per year without exceeding the 3% sludge limit allowable in its input fuel, but requires a dry matter content > 85% for acceptance, which can safely be reached only in summer or after more than 5-month processing in winter. Dried sludge could also be sent to the Zdzieszowice coking plant, 20 km away, which is part of ArcelorMittal Poland S.A. that is currently undergoing an expansion after which it could accept around 10,000 t/year of product in the future. In 2018, the plant used just 1125 t of dried sludge from various sources.

Gasification and co-combustion in power boilers may also be considered. Currently, however, co-incineration of sewage sludge is technically considered a process of waste destruction by Polish regulations, not renewable fuel recovery. This involves specific legal, technical and economic issues that hinder the feasibility of these solutions. Therefore, as long specific incentives and favourable regulations are introduced, it is debatable whether these sectors could be interested in this practice. Furthermore, municipal waste incineration plants in Poland are mostly built as facilities dedicated exclusively to municipal waste and do not contemplate acceptance of thermally (including solar dried) processed sewage sludge (Bień and Bień 2015).

Existing biomass digestion plants could accept partially dried sludge (e.g. 12–18-week “winter” sludge), as feedstock for co-digestion. This may be feasible especially for large facilities and could be considered after careful balance between higher transportation costs of the wetter sludge and energy recovery. An incentive in this case is that sludge would be treated as zero-emission material for the purpose of CO_2_ emissions reduction under this option. Residual sludge after methanation can be reused for co-incineration in biomass fuels mixtures with wood, straw or hard coal or sent to further processing for material recovery.

Wastewater sludge is a good source of nutrients, which is the main reason for its agricultural spreading. As a safer alternative to this practice, phosphorus could be recovered in mineral form from wastewater treatment processes, up to 90% of its influent amounts (Daneshgar et al. 2018b; Tomei et al. 2020). The Ujazd WWTP, due to its small size, is not suited for P recovery from the liquid treatment train and would require overly complex process modifications in exchange for low expected efficiency. However, other options are available: recovery may be achieved by chemical leaching from P-rich residual ashes after thermal processing of sludge (i.e. combustion, pyrolysis, gasification). Combination of thermal processing and phosphorus extraction from char residues could achieve 70–98% recovery efficiency (Atienza-Martinez et al. 2014). Another technology applicable to “wet” sludge (around 75–80% humidity, as achieved in winter after just 12–15 weeks) with low overall heating value is supercritical water gasification (SCWG) at high temperature (400–600 °C) and short process time (15–60 min). The process could recover > 95% P by leaching solids with acids (Acelas et al. 2014). Although interesting, this technology would be technically challenging in small facilities or would otherwise imply inefficient long range transfer of wet sludge to a suitable centralized plant.

After considering available options for disposal of the dried Ujazd WWTP sludge, the most sustainable could be conferring it to the Zdzieszowice coking plant (minimum transportation impact, residual value recovery), the second best to transport it to the Górażdże Cement Plant (longer distance, energy recovery mitigation), either one having ample capacity to accommodate this residue. Under current process conditions, however, either would imply at least 5-month winter process time, which would be feasible as long as sludge volumes remain at current levels. As an alternative, winter sludge could undergo a shorter process cycle (12–18 weeks) and sent to a nearby co-digestion facility, under a flexible disposal scheme according to seasonal handling capacity.

## Conclusions

Environmentally sustainable disposal of excess municipal sewage sludge is becoming a major focus of current Polish environmental policy. Unresolved issues concern mainly small and medium wastewater treatment facilities, which lack financial resources to build and operate advanced thermal treatment processes. Solar drying is a diffuse post-processing option in small WWTPs, requiring very low-energy input, reducing cost and environmental impact and making sludge transportation and storage affordable. Drying efficiency, however, depends on degree of irradiation and temperature, which vary throughout the year. In Polish climate conditions, greenhouse drying facilities cannot guarantee constant efficiency and process rates at the highest levels throughout the year, possibly precluding some of the possible options for final disposal in specific periods. Evaluation of all final disposal alternatives and adoption of multi-target, flexible disposal approaches could be an alternative to costly expansion or energetic augmentation of existing facilities.
